# Suicide Attempts, Neurocognitive Dysfunctions and Clinical Correlates in Middle-Aged and Elderly Chinese Schizophrenia Patients

**DOI:** 10.3389/fpsyt.2021.684653

**Published:** 2021-05-28

**Authors:** Yuanyuan Huang, Kai Wu, Rui Jiang, Xiaoying Zeng, Sumiao Zhou, Weijian Guo, Yangdong Feng, Caimei Zou, Hehua Li, Ting Li, Yuping Ning, Mingzhe Yang, Fengchun Wu

**Affiliations:** ^1^Department of Psychiatry, The Affiliated Brain Hospital of Guangzhou Medical University, Guangzhou, China; ^2^Department of Biomedical Engineering, School of Materials Science and Engineering, South China University of Technology (SCUT), Guangzhou, China; ^3^Qingdao Mental Health Center, Qingdao University, Qingdao, China; ^4^New Growth of Guangzhou, Guangzhou, China; ^5^The First School of Clinical Medicine, Southern Medical University, Guangzhou, China; ^6^Guangdong Engineering Technology Research Center for Translational Medicine of Mental Disorders, Guangzhou, China

**Keywords:** suicide attempts, neurocognitive function, schizophrenia, PANSS, middle-aged and elderly

## Abstract

**Background:** Suicide is a common and complex symptom of schizophrenia that may be related to clinical variables and neurocognitive function. This study aimed to investigate the associated correlates of suicide attempts in Chinese middle-aged and elderly inpatients with schizophrenia, including demographic and clinical characteristics and cognitive level, which has not yet been reported.

**Methods:** A total of 426 schizophrenia inpatients were recruited for this study. Clinical symptoms were evaluated using the Positive and Negative Syndrome Scale (PANSS). Neurocognitive function was measured by the Repeatable Battery for the Assessment of Neuropsychological Status (RBANS).

**Results:** The prevalence of suicide attempts in middle-aged and elderly Chinese schizophrenia patients was 13.3%. Female patients had a higher suicide rate than male patients. Patients with suicide attempts had significantly higher PANSS-positive subscores, depressive subscores, and RBANS-story recall than non-attempter patients (all *p* < 0.05). Multiple logistic regression showed that gender, positive subscore, depressive subscore and RBANS-story recall (OR = 1.10–2.19, *p* < 0.05) were independently associated with suicide attempts in middle-aged and elderly schizophrenia patients.

**Conclusions:** Our study showed that the rate of suicide attempts in Chinese middle-aged and elderly schizophrenia patients is high. Compared to non-attempters, there are less cognitive impairments, more clinical symptoms, and more female patients in the suicide attempters.

## Introduction

With the trend of an increased ageing population, the proportion of psychiatric disorders in middle-aged and elderly individuals has significantly increased. Schizophrenia is a serious mental disorder that has cognitive impairment, positive and negative symptoms and a high suicide rate (1). The prevalence of suicide attempts in schizophrenia patients ranges from 10 to 50% (2, 3), which is far more common (~10–30 times) than in the general population (4, 5). Suicide is considered to be the primary cause of shortening the life expectancy and worsening the prognosis of patients with schizophrenia (6, 7). The stress of caring for those with schizophrenia, especially in late-life schizophrenia (LLS), has become a major public problem (8), and ageing mental health patients are easy to ignore.

Risk factors for suicide attempts in schizophrenia patients are most relevant to demographic variables, clinical characteristics, and some treatment-related variables. A previous study investigated suicidal characteristics in 520 Chinese patients with schizophrenia and revealed that subjects who attempted suicide tended to be younger, single, have more severe depressive symptoms and longer hospital stays (2). Ran et al. (9) also reported that younger age, a higher rate of depressive symptoms, more positive symptoms and a higher level of education were positive factors for suicide attempts in schizophrenia. The age of onset of psychotic symptoms is independently associated with suicidal behaviour (3). Compared to those under the age of 20, young people between the ages of 30 and 39 have the highest risk of suicide (10). Gender is also a potential risk factor for suicidal behaviour in patients with schizophrenia. Uzun et al. (11) reported that the incidence of attempted suicide in men is higher than in women, and a nationwide cohort study with 174,039 patients demonstrated that women have a higher suicide rate due to jumping and drowning than men (10). Suicidal behaviour in the setting of schizophrenia is positively correlated with the presence of symptoms (1). Some studies showed that several clinical variables and sociodemographic factors are associated with suicidal behaviour in schizophrenia, but these results are often inconsistent (12), requiring more research.

Cognitive impairment is considered to be a cardinal feature of schizophrenia (13–15). However, the results on the relationship between suicide risk and cognitive impairment in schizophrenia have been largely mixed. Several studies have reported that higher cognitive functions in terms of verbal fluency, attention and cognitive flexibility may increase the risk of suicide in schizophrenia (16), while others have not identified a correlation between cognition and suicidal behaviours (17). These conflicting results may be related to different research methods, such as different locations of the study and the combination of neuropsychological tests and samples. Similarly, patients with mental disorders without a history of suicide also have cognitive impairment (18, 19), which illustrates the necessity of exploring whether the cognitive changes in patients with schizophrenia are related to suicidal behaviour. In addition, compared to the elderly healthy control group, there was obvious cognitive impairment in elderly patients with schizophrenia (20). At present, most studies have focused on the relationship between suicide attempts and cognition in schizophrenia without age distinction, and there are almost no research on middle-aged and elderly inpatients with schizophrenia.

To date, results of the correlation among suicide attempts and demographic data, clinical symptoms and cognition have been inconsistent, and there is no study on middle-aged and elderly Chinese inpatients with schizophrenia. To address the heterogeneity of suicidal tendencies in these inpatients, we collected detailed clinical characteristics and cognitive function assessed using detailed cognitive tools to determine (1) the incidence of suicide attempts in middle-aged and elderly Chinese schizophrenia inpatients; (2) whether patients with suicide attempts exhibit increased clinical symptoms; and (3) the relationship between neurocognition and suicide attempts in middle-aged and elderly Chinese schizophrenia inpatients.

## Methods

### Participants

The Institutional Review Board (IRB) of the Affiliated Brain Hospital of Guangzhou Medical University approved this study, and all participants provided informed consent to participate. Subjects were recruited from inpatients with schizophrenia at the Affiliated Brain Hospital of Guangzhou Medical University. All data were collected from February 2019 to September 2019. The recruitment criteria included those who (1) met DSM-IV diagnostic criteria of schizophrenia; (2) had at least 2 years of illness duration; (3) were aged ≥ 45 years, Han Chinese; and (4) received stable doses of antipsychotic drugs for at least 4 weeks before entering the study. The exclusion criteria were as follows: (1) the presence of severe somatic diseases, infectious diseases or immune system diseases; (2) pregnancy or lactation; (3) drug or alcohol abuse/dependence; (4) education level <5 years; and (5) ECT treatment within the last 6 months.

### Clinical Measurements

All participants completed detailed questionnaires, including general information, sociodemographic characteristics, history of suicide attempts, and psychiatric and medical history. In this study, patients who exhibited suicidal behaviour at least once in their lifetime were classified as suicide attempters, defined as a behavior of intentional self-harm without considering any consequences that did not cause death. Subjects who had not committed suicide in their lifetime were defined as non-attempters. Suicide attempts were assessed by a psychiatrist based on clinical interviews (all subjects were asked the question “did you ever attempt suicide at any time in your life?”), review of medical records, and confirmation with first-degree relatives and therapists if necessary.

All patients were assessed for psychopathological symptoms by two independent psychiatrists using the Positive and Negative Syndrome Scale (PANSS) (21, 22). Psychiatric scoring physicians participated in PANSS consistency training, and the correlation coefficient of the PANSS score was >0.8. Based on the five-factor PANSS model proposed by Wallwork et al. (23), clinical symptoms were divided into the following aspects: (1) negative factor: N1, N2, N3, N4, N6, and G7; (2) excited factor: P4, P7, G8, and G14; (3) positive factor: P1, P3, P5, and G9; (4) cognitive factor: P2, N5, and G11; and (5) depressed factor: G2, G3, and G6.

### Cognitive Assessments

The neurocognitive function of each participant was mainly evaluated by the Repeatable Battery for the Assessment of Neuropsychological Status (RBANS) (24) consisting of five domains of cognition. RBANS is made up of 12 subtests: (1) immediate memory: list learning and story memory; (2) language: picture naming and semantic fluency; (3) visuospatial/constructional ability: figure copy and line orientation; (4) delayed memory: list recall, list recognition, story recall, and figure recall; and (5) attention: forward digit span and coding. In this study, the scores of each test and the total RBANS scores were recorded. In addition, PANSS-cognitive factor (including P2: Concept Disorder, N5: Abstract Thinking Disorder and G11: Attention Disorder) in this study also reflect cognitive functions of patients with schizophrenia.

### Statistical Analysis

A sample test of Kolmogorov–Smirnov was applied to test for normal distribution. Regarding group differences between suicide attempters and non-attempters, continuous variables were tested by analysis of variance (ANOVA), and classified variables were analysed by the Chi-square test. Furthermore, analysis of covariance (ANCOVA) was performed to control for age, education, age at first hospitalization and sex as covariates. Multivariate logistic regression analysis (forward: conditional model) was conducted to check the risk factors of suicide attempters in middle-aged and elderly schizophrenia patients. Bonferroni correction was used in multiple tests. SPSS version 18.0 was used to perform all analyses, which have a significance level of 0.05 (*p* < 0.05).

## Results

### Demographic Characteristics

A total of 426 schizophrenic inpatients, including 146 females and 280 males, were recruited for this study. Their average age was 55.34 [standard deviation (SD) = 6.96]. Their duration of education was 9.11 years (SD = 3.15). The average age of onset was 25.34 (SD = 7.75), and the age at first hospitalization was 28.72 (SD = 10.31).

In the entire patient group, the prevalence of suicide attempts in those with schizophrenia was 13.3% (57/426), with rates of 19.18% (28/146) in female patients and 10.35% (29/280) in male patients, which represented a significant gender difference (X^2^ = 6.442, *p* = 0.016; Bonferroni corrected *p* < 0.05). There was also a significant difference in the age at first hospitalization (*p* < 0.05; Bonferroni corrected *p* > 0.05). All demographic data of patients in the non-attempter and suicide attempter groups are compared in Table 1.

**Table 1:** Demographic characteristics of attempter and non-attempter patients.

	**Non-attempters**	**Attempters**	**F/χ^2^**	***p-*value**
	***N* = 369**	***N* = 57**		
Age, years, M ± SD	55.35 ± 7.04	55.26 ± 6.47	0.007	NS
Gender, Male/female, *n*	251/118	29/28	6.442	0.016
Married, *n* (%)	91(24.66%)	13(22.81%)	1.104	NS
Education level, years, M ± SD	9.17 ± 3.14	8.72 ± 3.19	1.009	NS
BMI, kg/m^2^, M ± SD	24.41 ± 3.97	24.64 ± 3.79	0.167	NS
History of physical disease, *n* (%)	92 (24.93%)	14 (24.56%)	0.092	NS
Family history, *n* (%)	50 (13.55%)	6 (10.53)	0.395	NS
Smoking, *n* (%)	194 (52.57%)	27 (47.37%)	0.920	NS
Drinking, *n* (%)	108 (29.27%)	25 (43.85%)	7.534	NS
Age of onset, years, M ± SD	25.57 ± 7.89	23.189 ± 6.59	2.305	NS
Age of first hospitalization, M ± SD	29.14 ± 10.48	25.98 ± 8.61	4.67	0.031
Number of hospitalizations, M ± SD	6.65 ± 9.80	6.02 ± 8.05	0.038	NS
Antipsychotic drug dosage	329.80 ± 28.77	408.68 ± 74.59	1.011	NS
(CPZ equivalent mg), M ± SD				

*BMI, body mass index*.

### Clinical Variables in Suicide Attempters vs. Non-attempters

The suicide attempt patients had significantly higher PANSS-positive subscores, depressive subscores and excited subscores than non-attempter patients (all *p* < 0.05; Bonferroni corrected *p* < 0.05). When gender and age at first hospitalization were added as covariates, there were still significant differences in PANSS-positive subscores (F_1, 421_ = 2.763, *p* < 0.001, r^2^ = 0.142) and depressive subscores (F_1, 421_ = 4.083, *p* < 0.001, r^2^ = 0.142) between the two groups. There was no significant difference in negative and cognitive factors between suicide attempters and non-attempters (Table 1).

### Cognitive Function in Suicide Attempters vs. Non-attempters

The RBANS performance between suicide attempters and non-attempters was showed in Table 2. Compared to suicide attempters, non-attempters exhibited poorer cognitive performance in RBANS-list learning, semantic fluency and story recall (all *p* < 0.05; Bonferroni corrected *p* < 0.05). However, only RBANS-story recall (F_1, 421_ = 2.245, *p* = 0.009, r^2^ = 0.090) showed a significant difference after controlling for covariates, including gender, age, education level, and age at first hospitalization.

**Table 2:** Clinical variables and cognitive functions between attempters and non-attempters.

	**Non-attempters**	**Attempters**	**F**	***p-*value**
	***N* = 369, M ± SD**	***N* = 57, M ± SD**		
PANSS total score	75.62 ± 15.49	80.25 ± 16.16	4.322	0.038
Negative subscore	16.81 ± 5.34	18.33 ± 5.57	1.398	NS
Positive subscore	9.5 ± 3.87	12.35 ± 4.87	22.654	<0.001
Excited subscore	7.66 ± 2.88	8.75 ± 3.02	7.073	0.008
Depressive subscore	6.70 ± 2.43	8.81 ± 3.25	33.678	<0.001
Cognitive subscore	9.14 ± 2.82	9.21 ± 3.32	0.026	NS
RBANS total score	63.78 ± 12.50	66.37 ± 15.15	1.993	NS
Immediate memory	55.81 ± 29.56	60.32 ± 18.26	1.249	NS
List Learning	14.88 ± 6.21	17.23 ± 7.21	6.715	0.010
Story Memory	6.97 ± 4.63	7.96 ± 5.51	2.163	NS
Visuospatial/constructional	78.55 ± 16.71	79.51 ± 20.41	0.153	NS
Figure Copy	15.4 ± 4.72	15.26 ± 5.64	0.040	NS
Line Orientation	12.19 ± 4.43	11.74 ± 5.72	0.482	NS
Language	79.28 ± 12.93	79.04 ± 14.12	0.017	NS
Picture Naming	9.51 ± 4.96	9.21 ± 1.65	0.207	NS
Semantic Fluency	12.71 ± 4.91	14.53 ± 4.21	6.973	0.009
Attention	77.31 ± 14.73	78.60 ± 17.31	0.359	NS
Forward Digit Span	11.42 ± 4.05	11.39 ± 3.06	0.004	NS
Coding	22.28 ± 12.57	25.72 ± 13.12	3.654	NS
Delayed memory	62.94 ± 18.84	64.21 ± 21.10	0.218	NS
List Recall	2.16 ± 2.31	2.81 ± 2.61	3.759	NS
List Recognition	16.66 ± 9.74	16.72 ± 2.78	0.002	NS
Story Recall	3.32 ± 3.09	4.4 ± 3.67	7.747	0.007
Figure Recall	6.96 ± 8.47	6.81 ± 5.70	0.018	NS

*PANSS, positive and negative syndrome scale; RBANS, repeatable battery for the assessment of neuropsychological status*.

### Correlation of Suicide Attempts, Clinical Symptoms and Cognitive Performance

A multiple logistic regression model was used to explore relevant factors for suicide attempts in middle-aged and elderly inpatients with schizophrenia, taking suicide attempts as the dependent variable, and the independent variables were statistically significant factors in ANOVA (including gender, age of first hospitalization, PANSS total score, positive subscores, excited subscore, depressive subscores, RBANS-list learning, semantic fluency, and story recall). The results showed that gender (OR = 2.19; 95% CI: 1.19–4.054), positive subscore (OR = 1.12; 95% CI: 1.05–1.20), depressive subscore (OR = 1.24; 95% CI: 1.11–1.38) and RBANS-story recall (OR = 1.10; 95% CI: 1.00–1.21) were independent contributors to suicide attempts in schizophrenia (Table 3).

**Table 3:** Factors for suicide attempts in middle-aged and elderly schizophrenia patients.

	**Odds Ratio (OR)**	**95% CI**		***p*-value**
		**Lower**	**Upper**	
Gender	2.197	1.19	4.054	0.012
Positive subscore	1.122	1.047	1.203	0.001
Depressive subscore	1.236	1.105	1.381	<0.001
RBANS-Story Recall	1.103	1.005	1.21	0.038

*Variables in the model: Gender, Age of first hospitalization, PANSS total score, Positive subscore, Excited subscore, Depressive subscore, RBANS-List Learning, Semantic Fluency and Story Recall*.

## Discussion

To our best knowledge, we first explored the correlation between suicide attempts and clinical symptoms as well as neurocognitive function in middle-aged and elderly Chinese schizophrenia inpatients. Our main findings are as follows: (1) the prevalence of suicide attempts was 13.3% in middle-aged and elderly schizophrenia patients. (2) Patients with suicide attempts performed better on RBANS-list learning, semantic fluency and story recall than those without suicide attempts. (3) Females are more likely to be suicide attempters than males. (4) PANSS-positive symptoms and depressive symptoms are independently associated with suicide attempts.

In our study, the proportion of suicide attempts (13.3%) in middle-aged and elderly schizophrenia patients was similar to the results of the study of chronic schizophrenia patients with a larger age range (such as 18–65 years old, 25–75 years old, etc.) in China (9.2 and 12%) (2, 25). Interestingly, reported suicide rates in patients with schizophrenia vary widely. For example, a study involving 510 outpatients with schizophrenia found that the incidence of suicide attempts in rural patients was 7.5% (9). Xiang et al. conducted a study of 505 schizophrenia outpatients and found that 26.7% of patients had a history of suicide attempts in their lifetime, with rates of 20% in Hong Kong and 33.6% in Beijing (26). In regard to other countries, a current review reported that 10–50% of schizophrenia patients reported a history of suicide attempts (3). The reasons for the differences in suicide rates, especially those domestically and abroad, might be due to differences in research samples (including cultural background, treatment environment, ethnic genes, etc.) (2), sampling methods, and the assessment methods used for suicide attempts (25), which requires an epidemiological study with more samples and a broader geographic area in the future to provide stronger evidence.

Our main findings regarding suicide attempts and neurocognitive function supported the results of previous studies (16, 17, 27), which found that better cognitive performance might increase suicide risk. A recent study on cognition and suicide attempts found that cognitive planning ability is independently related to suicide attempts in schizophrenia patients (28). However, differences are presented in the cognitive assessment tools and the methods used to evaluate suicide in these studies. In this study, we used two instruments to evaluate the cognitive function of patients. A preliminary exploration of cognitive function in the PANSS rating scale did not find a significant correlation between cognitive factors and attempted suicide. However, the more comprehensive and popular RBANS cognitive test found that patients with suicide attempts performed better on list learning, semantic fluency and story recall than those without suicidal behaviour, and RBANS-story recall in the delayed memory domain was an independent influencing factor of suicidal behaviour. Delaney et al. (27) found that patients with suicidal ideation and single suicide attempts outperformed patients with no suicidal behaviour or ideation on global cognition (measures of IQ). A study of 333 patients examined by a neurocognitive battery reported an association between better cognitive performance and greater suicidality in schizophrenia (29). These previous studies concluded that there is an association between suicide attempts and different cognitive domains, such as attention, planning ability and execution ability which may be related to differences in research methods, especially the cognitive evaluation tools and the methods used to evaluate suicide (18). However, all of these results suggest that higher cognitive scores may represent a risk factor for suicide. Furthermore, some studies have revealed that suicide attempts are a conscious initiation of goal-oriented behaviour (30). Better executive function (31) and attention skills (16) may enable patients to plan and initiate targeted goals of suicidal behaviours, which may increase the impulse to commit suicide. To date, there was no study on suicide and cognition in middle-aged and elderly schizophrenia patients. We found that there were less cognitive impairments in the suicide attempters than non-attempters. Similar to a study in older depressed adults, Arslanoglou et al. (32) reported that subjects with suicidal ideation had higher memory scores than those without suicidal ideation. In contrast, our results did not replicate those of other previous neurocognitive studies (33–35), which revealed no relationship between suicide and cognition. A recent review indicated that patients with suicidal behaviour have poorer executive function performance than those without suicidal behaviour (35). A cross-sectional study in 316 chronic inpatients with schizophrenia conducted in Beijing, China found that performance on the total and index RBANS test was not significantly associated with lifetime risk of suicide attempts (33). In summary, there may be an internal relationship between suicide attempts and neurocognition in middle-aged and elderly schizophrenia patients, but the results are superficial and inconsistent and need to be confirmed by further prospective studies in larger samples.

Another interesting finding was that female patients with schizophrenia had a higher suicide attempt rate than male patients, which was consistent with previous studies (36, 37). However, other studies have reported that a higher risk of suicidal behavior in men with schizophrenia (38, 39). Chun-Hung et al. (10) even indicated that although the suicide rate in female patients with schizophrenia was lower than that in male patients, their standardized mortality ratio was higher than that in male patients. Therefore, patients with schizophrenia of any gender need to be monitored for suicidal behaviour to strengthen prevention.

More importantly, we identified significantly positive associations between clinical symptoms and suicide attempts, including the clinical symptoms of PANSS positive symptoms and depressive symptoms being risk factors for suicidal behaviour, which confirmed and extended most previous studies on suicide attempts in patients with schizophrenia (1, 11, 25, 28, 40). A review by Avinash et al. that summarized articles from 2015 to 2019 concluded that positive symptoms played an important role in suicide in schizophrenia patients (1). Similar to Yan et al. report of suicides in schizophrenia (25), a study that investigated the clinical characteristics and symptoms of suicide in patients with schizophrenia in Taiwan showed that depressive and psychotic symptoms were contributing factors to suicidal behaviour (41). Xiang et al. also revealed that Chinese schizophrenia patients who attempted suicide tend to have more severe anxiety and positive depressive symptoms (26). Therefore, current studies indicate that the more severe the clinical symptoms, the more obvious the suicidal tendency, which is the same as that observed in middle-aged and elderly schizophrenia patients. Moreover, patients have more positive symptoms, including psychotic symptoms such as auditory delusions and hallucinations, which make patients more motivated to plan and initiate suicidal behaviour. On the other hand, the relationship between suicide and depression in schizophrenia is complex. Depression may be a symptom group of schizophrenia, which can increase suicide rate in the following year and lifetime suicidal behaviour (42); however, depression can also be diagnosed separately (43). Regardless of the symptom connection, it is necessary to guard against suicidal tendencies in patients with schizophrenia through timely evaluation and intervention.

Several limitations should be noted in this study. First, limited to the current definition of attempted suicide, we only evaluated whether there were suicide attempts, which lacked specific grading assessment of the degree of suicide (such as suicidal ideation) and lacked examination of specific methods to induce suicide. Second, a cross-sectional study did not directly illustrate a causal relationship between suicide attempts and risk factors in middle-aged and elderly Chinese inpatients with schizophrenia. Third, there was no healthy control group in the current study. Fourth, the subjects included in our study were long-term inpatients who had a longer course of disease, more severe symptoms and longer drug treatment than first-episode or outpatient patients, which may affect neurocognitive outcomes. Fifth, the sample size of the suicide attempters group and the non-attempters group appeared uneven, which might have a certain impact on the results. Therefore, our current survey results are only preliminary and need to be confirmed by prospective studies with a larger sample size in the future.

In conclusion, our results extend previous reports and identified a high incidence of suicide attempts in middle-aged and elderly Chinese schizophrenia patients. Female patients were more likely to be suicide attempters than male patients. Compared to non-attempters, there were less cognitive impairments and more clinical symptoms in the suicide attempters. PANSS-positive symptoms, depressive symptoms and RBANS-story recall were associated with suicide attempts in middle-aged and elderly Chinese schizophrenia patients. Although suicide in psychiatric patients is caused by multiple factors and the cross-sectional study cannot explain the causal association between suicide attempts and schizophrenia, timely detection and intervention will greatly reduce the incidence of suicide. Future prospective researches with large sample sizes are required to further explain the potential risk factors for suicide attempts in middle-aged and elderly schizophrenia patients.

## Data Availability Statement

The original contributions presented in the study are included in the article/supplementary material, further inquiries can be directed to the corresponding authors.

## Ethics Statement

The studies involving human participants were reviewed and approved by the Institutional Review Board (IRB) of the Affiliated Brain Hospital of Guangzhou Medical University. The patients/participants provided their written informed consent to participate in this study.

## Author Contributions

FW and MY: were responsible for management and oversight of the study. YH and KW: were responsible for general omnibus data analyses and were keys contributing authors to the manuscript. XZ, HL, and YF: were responsible for all research interviews and clinical chart reviews associated with this study. TL and YN: provided guidance on the design of primary analyses. RJ and SZ assisted with all data collection, analysis, and writing of the manuscript. All authors contributed to the study design, data interpretation, critically reviewed the manuscript, and gave final approval for its publication.

## Conflict of Interest

The authors declare that the research was conducted in the absence of any commercial or financial relationships that could be construed as a potential conflict of interest.
